# *Madurella Mycetomatis* Infection Following Allogenic Stem Cell Transplantation for Aplastic Anemia

**DOI:** 10.4084/MJHID.2012.038

**Published:** 2012-06-14

**Authors:** Sanjeev Kumar Sharma, Anjan Mukherjee, Avinash Kumar Singh, Tulika Seth, Suman Kumar, Pravas Mishra, Immaculata Xess, Somesh Gupta, Manoranjan Mahapatra, Haraprasad Pati

**Affiliations:** 1 Department of Hematology, All India Institute of Medical Sciences, New Delhi, India; 2 Department of Microbiology, All India Institute of Medical Sciences, New Delhi, India; 3 Department of Dermatology, All India Institute of Medical Sciences, New Delhi, India

**Dear Editor,**

Eumycetoma is a slow growing fungal infection, which initially involves the skin and subcutaneous tissues and progresses to involve deeper structures. It is characterized clinically by multiple sinuses draining purulent exudate containing fungal granules. Since the foot is the most common site of initial localization of the lesion, the term “Madura foot” has been used to describe it. Strong clinical suspicion followed by categorization of lesion into eumycotic or actinomycotic by suitable culture is essential for diagnosis and effective therapy as delay in diagnosis may be limb or life threatening. We report here, a case of madura foot caused by *Madurella mycetomatis,* in a patient with aplastic anemia following allogenic peripheral blood stem cell transplantation with favourable clinical outcome.

A 22-years-old male was diagnosed as a case of very severe aplastic anaemia seven months back when he presented with fever and gum bleeding. He had HLA matched sibling and underwent allogenic peripheral blood stem cell transplantation. Conditioning regimen included fludarabine 30mg/m^2^ for 6 days and cyclophosphamide 60mg/m^2^ for 2 days. Graft versus host disease (GVHD) prophylaxis included anti-thymocyte globulin 30mg/kg/day for 4 days, methotrexate (10mg/m^2^) on day +1, +3 and +6, and cyclosporine 100mg twice daily intravenously followed by oral cyclosporine with dose adjusted according to plasma cyclosporine levels (between 150–300ng/ml). He engrafted on day +9 (absolute neutrophil count >0.5×10^9^/l and unsupported platelet count > 20×10^9^/l). Two months post transplant patient noticed a small painless nodular swelling over the plantar surface of right foot. He was on cyclosporine at that time. There was no history of trauma or thorn prick injury. The swelling progressively increased in size in next two months with appearance of two discharging sinuses (Figure 1). Radiograph of right foot revealed soft tissue radio-opacity without involvement of underlying bony structures. There was discharge of black grains which on KOH mount examination under microscope (40× magnification) revealed presence of septate hyphae. The culture of the discharged granules in Sabouraud’s dextrose agar (SDA) grew *Madurella mycetomatis* (Figure 2) after an incubation for 12 days. The patient was started on voriconazole (400 mg orally twice daily on first day followed by 200 mg twice daily). The lesion was completely excised one week later. Biopsy smears showed distinct brown-black colonies of the fungus having branching and septate hyphae embedded in matrix like material against a mixed inflammatory background. Voriconazole was continued and there is no recurrence of lesion three months post excision.

With the increasing number of patients undergoing transplantation, newer fungal infections are emerging and posing a diagnostic and therapeutic challenge. Mycetomatis is a chronic infection which initially involves cutaneous areas and progresses to involve deeper structures. Mycetoma presents as a slow growing indolent infection characterised by tumaefaction and multiple discharging sinuses. The etiological agents can be a variety of fungal agents (eumycetoma) or bacteria belonging to the actinomycetes group (actinomycetoma). *Madurella mycetomatis* is the worldwide predominant eumycetoma, followed by *Scedosporium apiosperma, Scedosporium prolificans,* and *Madurella grisea*.^1^ These fungi account for approximately 95% of eumycetoma cases. *Madurella mycetomatis* (black grain mycetoma) is the most common eumycetoma in India.^2^,^3^ It is also endemic in Sudan, Central and South America and Indonesia.^4^ In contrast, Scedosporium (white grain mycetoma) is the commonest eumycetoma in North America.^5^ It is the most commonly reported eumycetoma in transplant recipients, which is associated with a high rate of dissemination and a high mortality.^5^ There is no data of madurella infection in transplant recipients except for two reported cases who received immunosuppressive therapy for renal transplantation (infected by *Madurella grisea and Madurella mycetomatis*, respectively).^6^,^7^ With the increasing number of patients undergoing transplants in tropical and subtropical countries, *Madurella mycetomatis* will be an emerging fungal infection in such a group of population.

The differential diagnosis of eumycetoma lesions include actinomycetoma, botryomycosis, cutaneous tuberculosis, fungal diseases such as blastomycosis and coccidioidomycosis, and cutaneous nocardiosis. An early diagnosis is essential since the disease has a progressive course with extension and damage to deeper tissues and internal organs, particularly more so in immunocompromised patients. Discharging sinus tracts are very characteristic of mycetoma and are helpful in making the clinical diagnosis. The cytological diagnosis of eumycetoma can be as accurate as histological diagnosis, and techniques such FNAC as well as imprint smears are simple, inexpensive and fairly reliable techniques without any obvious disadvantages.^8^

Early surgery can be curative for a small, localized eumycetoma lesion amenable to total excision and has been considered as a standard of care for such patients.^9^ Medical therapy of nonresectable or disseminated disease in immunocompromised patients is ineffective^9^ and may require aggressive debridement. Ketoconazole has been found effective for mycetoma caused by *M. mycetomatis* in at least 70% cases but is not an effective treatment for eumycetoma caused by *Scedosporium* species. The new second generation triazoles, including voriconazole and posaconazole have been tried as potential agents for eumycetoma, with some success.^10^,^11^

Our patient developed nodular lesion on the plantar surface of right foot two months post transplant which slowly progressed and resulted in discharging sinuses. Biopsy revealed infection by *M. mycetomatis*. This is probably the first reported case of *M. mycetomatis* infection in a patient following allogenic hematopoietic stem cell transplantation. Early complete excision of the lesion resulted in complete cure. With increasing number of patients undergoing stem cell transplantation in tropical countries, eumycetoma caused by *M. mycetomatis* is one of the emerging fungal infections among transplant recipients, because of prolonged duration of immunosuppression. Since the fungus has poor response to antifungal drugs, early surgical debridement is very essential before the infection spreads to involve deeper structures.

## Figures and Tables

**Figure 1:**
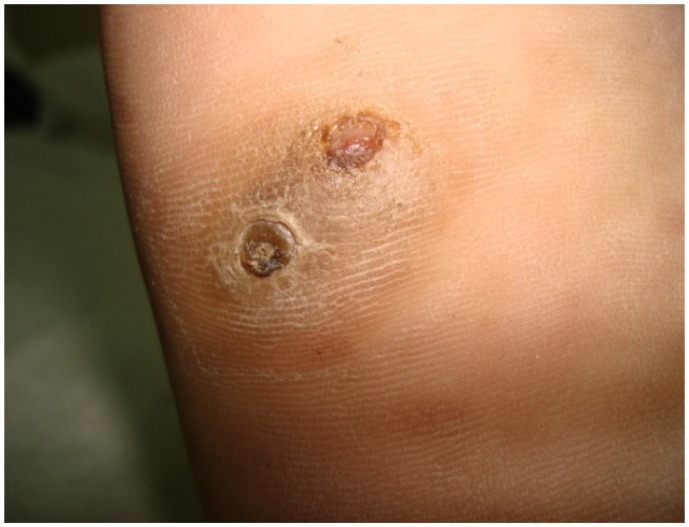
Lesion on the foot showing two discharging sinuses.

**Figure 2:**
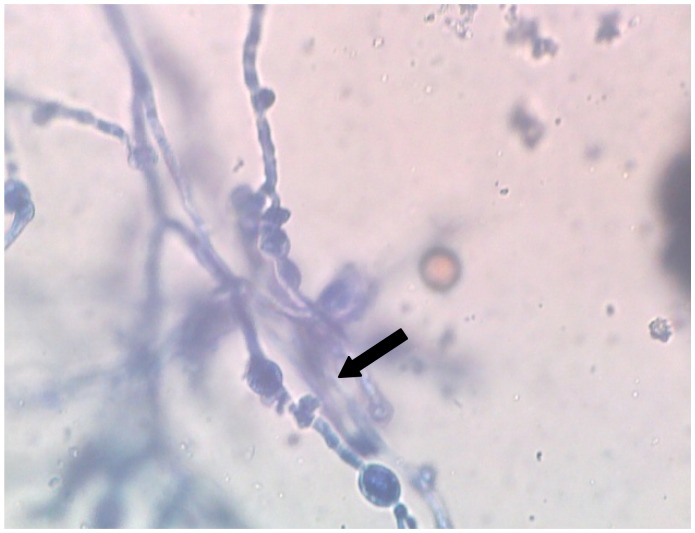
Wet mount from culture stained with Lactophenol Cotton Blue (LCB) showing brown pigmented septate hyphae with intercalary chlamydospores (arrow) characteristic of *Madurella mycetomatis.*
